# Corticotherapy versus adrenocorticotropic hormone for treating West syndrome: a systematic review and meta-analysis

**DOI:** 10.1055/s-0045-1811173

**Published:** 2025-08-31

**Authors:** Elizabeth Honorato de Farias, Anderson Matheus Pereira da Silva, Karlos Daniell Araújo dos Santos, Altair Pereira de Melo Neto, Aishwarya Koppanatham, Júlia Cappi Aguiar Moraes Souza, Niels Pacheco-Barrios, Gabrielle de Souza Rocha, Gustavo Sousa Noleto

**Affiliations:** 1 Universidade Federal de Roraima, Faculdade de Medicina, Boa Vista RR, Brazil.; 2 Universidade Federal do Vale do São Francisco, Faculdade de Farmácia, Petrolina PE, Brazil.; 3 Andhra Medical College, Faculty of Medicine, Visakhapatnam, Andhra Pradesh, India.; 4 Universidade de Rio Verde, Faculdade de Medicina, Rio Verde GO, Brazil.; 5 Brigham and Women's Hospital, Department of Neurosurgery, Boston MA, USA.; 6 Universidade Federal do Piauí, Teresina PI, Brazil.

**Keywords:** Spasms, Infantile, Epilepsy, Adrenocorticotropic Hormone, Adrenal Cortex Hormones

## Abstract

**Background:**

Although it is known that the most commonly used therapies for West syndrome (WS) are intramuscular adrenocorticotropic hormone (ACTH) and oral prednisolone, there is still controversy in the literature regarding the equivalence of their effects.

**Objective:**

We aimed to present an updated review comparing the therapeutic and adverse effects of ACTH therapy versus corticosteroids in children with West syndrome (WS).

**Methods:**

The PubMed, EMBASE, and Cochrane Central databases were searched. The outcomes of interest selected were spasm cessation on day 14 of therapy, cessation of hypsarrhythmia and adverse effects such as weight gain, infection, irritability, and hypertension. Studies were reviewed in accordance with the Preferred Reporting Items for Systematic Reviews and Meta-Analyses (PRISMA) guidelines, and a meta-analysis was performed. We compared the results using the risk ratio (RR) and odds ratio (OR). for the binary outcomes, with 95% confidence intervals (CI) and a random-effects model. Statistical analysis was performed using RevMan 5.1.7.

**Results:**

Compared with corticoids, ACTH was associated with a significant increase in weight (RR: 1.41; 95% CI: 1.01–1.97; *p* = 0.04). There was no significant difference in cessation of spasms on day 14 (OR: 0.91; 95% CI: 0.47–1.47; *p* = 0.79), hypsarrhythmia (OR: 0.97; 95% CI: 0.22–4.34), irritability (RR: 0.78; 95% CI: 0.30–1.99), hypertension (RR: 0.64; 95% CI: 0.35–1.15; *p* = 0.14), and infection (RR: 0.69; 95% CI: 0.19–2.50; *p* = 0.57).

**Conclusion:**

This study provides robust evidence regarding the safety and efficacy of ACTH or corticoids in children with WS. However, the significant heterogeneity between studies restricts the analysis, emphasizing the need for additional research to assess the best WS treatment option.

## INTRODUCTION

West syndrome (WS) is an epileptic encephalopathy characterized by the triad of epileptic spasms, hypsarrhythmia on electroencephalography (EEG), and developmental regression. 1 It was first described in 1841 by Dr. William James West in a letter to *The Lancet* . 2 The etiology of WS is diverse, encompassing genetic, structural, metabolic and idiopathic causes. Despite the wide range of potential triggers, the precise mechanism responsible for the generation of spasms remains unclear. The most accepted theory suggests that spasms are caused by excessive release of corticotropin-releasing hormone (CRH) in the brain, which has known excitatory and convulsive properties. 3 However, the relationship between different causal factors and the underlying pathophysiology of the syndrome is still not fully understood. 1

Treatment for WS has significant clinical challenges, commonly employed therapies include adrenocorticotropic hormone (ACTH), oral corticosteroids such as prednisolone, and anticonvulsants like vigabatrin. 4 This hormone is widely regarded as the first-line treatment, however it is associated with high costs, the necessity for hospitalization, and considerable adverse effects. Oral corticosteroids, particularly prednisolone, present advantages in terms of cost and route of drug administration, though there is ongoing debate regarding their comparative efficacy to ACTH. Vigabatrin is an alternative treatment but carries the risk of irreversible visual impairment. 1

Nevertheless, opinions regarding the relative merits of these treatments have varied over time. Early research indicated that ACTH and corticosteroids were equally effective, but later studies proposed the first might achieve a faster cessation of spasms. Despite the widespread use of ACTH, its limitations have prompted investigations into alternative therapies such as prednisolone. 1 5

Despite the existing treatments, it remains unclear which therapeutic option—ACTH or corticosteroids—is the most effective and safest first-line therapy for WS, particularly when considering cost and convenience of administration in different healthcare settings. Given the substantial financial and logistical burden associated with ACTH and the potential efficacy of oral corticosteroids like prednisolone, there is an urgent need to clarify the role of corticosteroids in the management of WS. If prednisolone proves to be equally effective or superior to ACTH, it could represent a valuable alternative, particularly in resource-limited settings where the cost and condition of ACTH delivery are prohibitive.

This article presents a systematic review and meta-analysis aimed at comparing the efficacy and safety of corticosteroid therapy versus ACTH for the treatment of WS children, focusing on spasms cessation and side effects incidence.

## METHODS

This systematic review and meta-analysis followed the guidelines established by the Cochrane Handbook and the Preferred Reporting Items for Systematic Reviews and Meta-Analyses (PRISMA) 2020 statement. 6 7 The protocol was prospectively registered on PROSPERO, under number: CRD42024596237.

### Search strategy and data extraction

The research presented in this systematic review and meta-analysis followed the PRISMA guidelines. 8 We searched the PubMed, Embase and Cochrane Library databases from July to August 2024. The following terms were used, combined with Boolean operators (“AND” and “OR”): *infantile spasm* , *West syndrome* , *epileptic spasm* , *epilepsy* , *infantile epileptic spasms* , *adrenocorticotropic hormone* , *ACTH* , *Corticotrophs* , *steroids* , *corticosteroids* , *prednisolone* , *methylprednisolone* , *Randomized Controlled Trial* and *RCT* .

### Eligibility criteria

#### *Inclusion criteria*

The inclusion criteria were randomized controlled trials (RCTs) or therapeutic studies about patients diagnosed with infantile spasms; treated with ACTH, prednisolone, methylprednisolone, or tetracosactide; compared with alternative treatment groups within the included studies; who seizure cessation, reduction in seizure frequency, electroencephalographic improvement, or adverse effects at the end of their studies.

There were no restrictions on the publication date of the included studies.

#### *Exclusion criteria*

The exclusion criteria were studies with less than 20 participants. Those with data reanalysis considering only standardized interventions in terms of corticosteroid or ACTH dosage and formulation. Also, those with comparison between fixed-effect and random-effects statistical models to evaluate the influence of heterogeneity. Finally, studies with separate analyses of each primary outcome on the overall results.

These approaches enhanced the reliability and consistency of the findings.

### Selection studies

The articles were uploaded to the Rayyan ( https://www.rayyan.ai/ ) platform 8 for title and abstract reading. In this stage, all articles that did not directly address the subject of interest were excluded, as were duplicates. Two reviewers performed this stage independently; any doubts were clarified jointly between them.

### Data extraction

The following data were extracted from the selected articles: authors, year of publication, study location, type of study, sample size and age, patient characteristics, duration of intervention, therapeutic scheme, follow-up time, main variables and main results. Two reviewers were responsible for extracting and managing the data independently, which were inserted into an Excel (Microsoft Corp.) spreadsheet; doubts were clarified with the help of the third researcher.

### Endpoints and sensitivity analyses

The endpoints of interest included spasm cessation, spasm recurrence, treatment-related adverse effects, EEG normalization, and need for treatment modification.

Spasm cessation was defined as the complete absence of epileptic spasms, either reported by caregivers or confirmed by video-EEG monitoring. Recurrence was considered as the reappearance of spasms after an initial phase of control. Treatment-related adverse effects were hypertension, weight gain, irritability, gastrointestinal disturbances, and increased risk of infection. Normalization of EEG was defined as the resolution or significant improvement of hypsarrhythmia patterns. The need for treatment modification was determined by the necessity to switch from corticosteroids to ACTH (or vice versa) due to treatment failure or intolerable adverse effects.

### Quality assessment

The risk of bias for each included study was evaluated using the revised Cochrane Risk of Bias (RoB2) tool for randomized trials, 9 which categorizes studies as having a high, low, or unclear risk of bias. Two authors independently performed this assessment, and any disagreements were resolved by consensus with a third author.

### Statistical analysis

We computed pooled odds ratio (OR) with 95% confidence intervals (CIs) were used to compare the effects of treatment on the cessation of spasms on day 14 and hypsarrhythmia. The outcomes of hypertension, infection, irritability and weight gain were assessed using the risk ratio (RR) with 95% CIs. We assessed heterogeneity with I ^2^ statistic; *p* -value < 0.10 and I ^2^ > 25% were considered significant for heterogeneity. 7 We used the Laird random-effects model and used. This was performed using Review Manager (RevMan 5.4) software (The Cochrane Collaboration) for statistical analysis.

Sensitivity analyses were conducted for all evaluated outcomes to ensure the robustness of the findings. The s0ubgroup analyses explored potential sources of heterogeneity, such as study characteristics and variations in intervention protocols. Statistical analysis was performed using R (R Foundation for Statistical Computing), version 4.3.2, with the meta package. 10 The Leave-One-Out method was applied to assess the impact of individual studies on the overall results and to identify potential outliers.

## RESULTS

### Study selection

**Figure 1:**
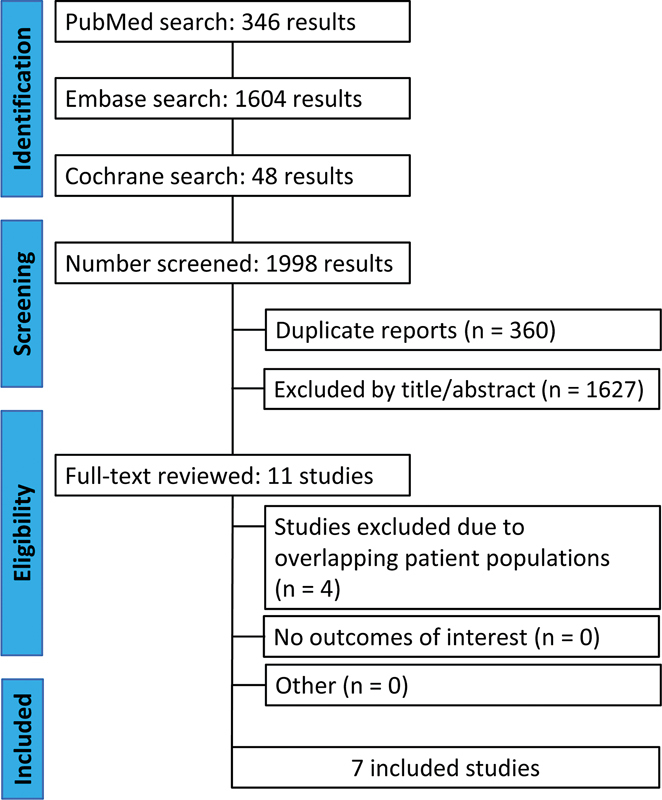
PRISMA flow diagram.

The initial search yielded 1,998 results from three databases: 346 from PubMed, 1,604 from Embase, and 48 from Cochrane. These records underwent an initial screening. After removing 360 duplicates, 1,638 records remained. Based on title and abstract evaluation, 1,627 studies were excluded, leaving 11 articles for full-text eligibility assessment. After full-text review, 4 studies were excluded due to overlapping patient populations. None were excluded for lacking relevant outcomes or other reasons. Ultimately, 7 RCTs 1 2 3 4 5 11 12 met the inclusion criteria. The PRISMA flow diagram is shown in Figure 1 , detailing the reasons for the exclusion of full-text articles.

### Baseline characteristics

There were 7 studies with a total of 426 patients, of which 215 (50.5%) received CT and 211 (49.5%) treated with ACTH. The mean age of patients in the CT group was 9.87 ± 2.14 months, while in the ACTH group, it was 8.45 ± 2.09 months. Among the CT patients, 8 were male and 117 were female, whereas in the ACTH group, 94 were male and 117 were female.

Corticosteroid therapies included prednisolone, methylprednisolone, and dexamethasone, while ACTH was administered in various dosing regimens and tetracosactide, an analog of the natural hormone, was used for comparison. 11 The inclusion criteria across studies focused on children diagnosed with WS, confirmed through clinical and EEG findings, while exclusion criteria varied, often involving comorbid conditions such as tuberous sclerosis, prior steroid exposure, or significant organ dysfunction. Detailed characteristics of the included studies are presented in Table 1 .

**Table 1:** Characteristics of the studies included in the current systematic review, including baseline patient information

Study, year	Study design	Country	Patients (n)	ACTH/CT (n)	Sex (M/F)	Mean age †	Inclusion criteria	Exclusion criteria
Baram, 1996 3	RCT	USA	29	15 ACTH 14 prednisone	NR	ACTH: 5.1 Prednisone: 7.5	Infantile spasms, hypsarrhythmic EEG, no prior steroid/ACTH treatment	Severe hypertension, resolution of spasms after shunt placement
Fatema, 2021 12	RCT	Bangladesh	75	43 ACTH 32 methylprednisolone	Methylprednisolone: 22/10 ACTH: 27/16	Methylprednisolone: 13.91 (6.234) ACTH: 11.63 (6.321)	Children aged 3–24 months with WS	Tuberous sclerosis, liver/kidney disorder, infection, electrolyte imbalance
Gowda, 2018 2	RCT open-label	India	34	18 ACTH 16 prednisolone	Prednisolone: 9/7 ACTH: 12/6	Prednisolone: 13.9 ACTH: 9.4	Children aged 2 months to 5 years with WS	Previous steroid treatment, tuberous sclerosis, contraindications for steroids
Hrachovy, 1983 5	RCT (Crossover)	USA	24	12 ACTH 12 prednisone	NR	3.5 to 24	Infantile spasms, hypsarrhythmic EEG	NR
Osborne, 2023 11	RCT	UK Australia Germany NZ Switzerland	126	64 prednisolone 62 tetracosactide	NR	NR	Aged 2–12 months (UKISS), 2–14 months (ICISS) with infantile epileptic spasms	Previous treatment with trial medications, tuberous sclerosis, pyridoxine dependency
Rajpurohit, 2020 4	RCT	USA	44	26 ACTH 18 methylprednisolone	31/13	Methylprednisolone: 11.9 (3–24)	Children aged 3 months to 3 years with WS, excluding TSC	TSC, parental refusal
Wanigasinghe, 2015 1	RCT	Sri Lanka	97	49 ACTH 48 prednisolone	56/41	Prednisolone: 8.31 ACTH: 9.93	Newly diagnosed patients with infantile spasms, confirmed diagnosis through observation	NR

Abbreviations: ACTH, adrenocorticotropic hormone; CT, corticotherapy; EEG, electroencephalogram; ICISS, International Classification of Diseases Injury Severity Score; n, number; NR, not reported; RCT, randomized clinical trial; TSC, tuberous sclerosis complex; UKISS, United Kingdom Infantile Spasms Study; WS, West syndrome; y, years.

Note: † are expressed as mean (SD) or median (range).

### Pooled Analysis of Included Studies

#### *Efficacy outcome*

**Figure 2:**
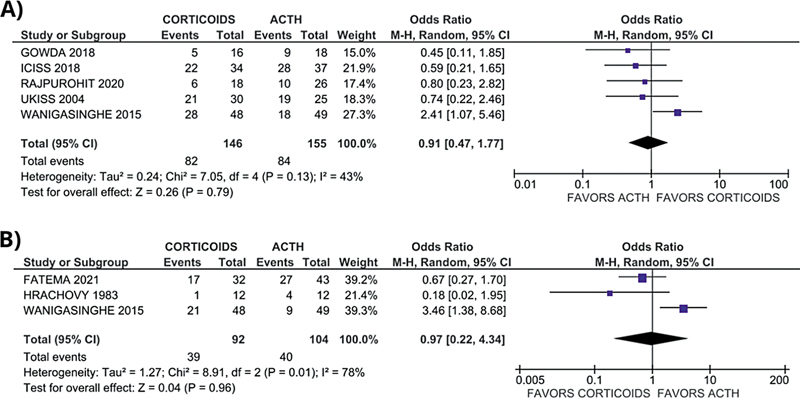
Forest plots illustrating the pooled OR for clinical outcomes in comparisons of corticosteroids versus ACTH in children with WS. With the OR for ( **A** ) spasm cessation and ( **B** ) reduction of hypsarrhythmia.

There was no statistically significant improvement in the cessation of spasms on day 14 in the ACTH group compared with corticosteroids (OR: 0.91; 95% CI: 0.47–1.47; *p* = 0.79; I ^2^ = 43%), as shown in Figure 2A . However, no significant difference was observed in the occurrence of hypsarrhythmia between the ACTH and corticosteroid groups (OR: 0.97; 95% CI: 0.22–4.34; I ^2^ = 0%) as shown in Figure 2B .

#### *Safety outcomes*

**Figure 3:**
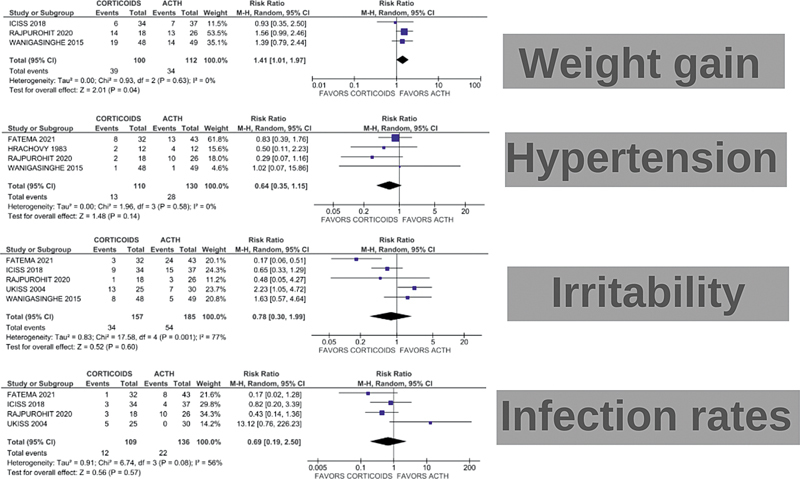
Forest plots illustrating RR for adverse effects in WS children treated with corticosteroids versus ACTH. The plots represent the outcomes for ( **A** ) weight gain, ( **B** ) hypertension, ( **C** ) irritability, and ( **D** ) and infection rates.

During the 2 to 6-week treatment period, patients receiving ACTH showed a statistically significant increase in weight gain compared with those taking corticosteroids (RR: 1.41; 95% CI: 1.01–1.97; *p* = 0.04; I ^2^ = 0%). Hypertension outcomes, measured across 4 studies, showed no significant improvement in the ACTH group (RR: 0.64; 95% CI: 0.35–1.15; *p* = 0.14; I ^2^ = 77%). Additionally, there was no significant improvement on irritability (RR: 0.78; 95% CI: 0.30–1.99; I ^2^ = 77%, or infection rates (RR: 0.69; 95% CI: 0.19–2.50; *p* = 0.57; I ^2^ = 56%) in patients treated with ACTH compared with the corticosteroid group, as shown in Figure 3A–D .

### Sensitivity analysis

The robustness of the pooled estimates was assessed through sensitivity analyses using the Leave-One-Out technique **Supplementary Material** ( https://www.arquivosdeneuropsiquiatria.org/wp-content/uploads/2025/08/ANP-2025.0023-Supplementary-Material.docx ), as shown in Figure S2 (online only). Studies were sequentially omitted.

The overall OR for spasm cessation ranged from 0.78 to 1.23, with broadly overlapping confidence intervals, indicating consistency in the results. Following the sensitivity tests, the pooled OR was 0.84 (95% CI: 0.41–1.74), with no substantial changes to the conclusions. Heterogeneity, assessed using the I ^2^ , ranged from 0 to 62%, indicating moderate variability among studies but without significant impact on the interpretation of the results. The Influence Plot ( **Supplementary Material Figure S3** , online only) shows that most studies had minimal impact on heterogeneity and the effect estimate. The Wanigasinghe et al. 1 study showed a slight influence but did not alter the direction or significance of the overall effect. Sensitivity analyses confirmed the robust results, with consistent estimates even in the presence of moderate heterogeneity, in some studies.

### Risk of bias of included studies

**Figure 4:**
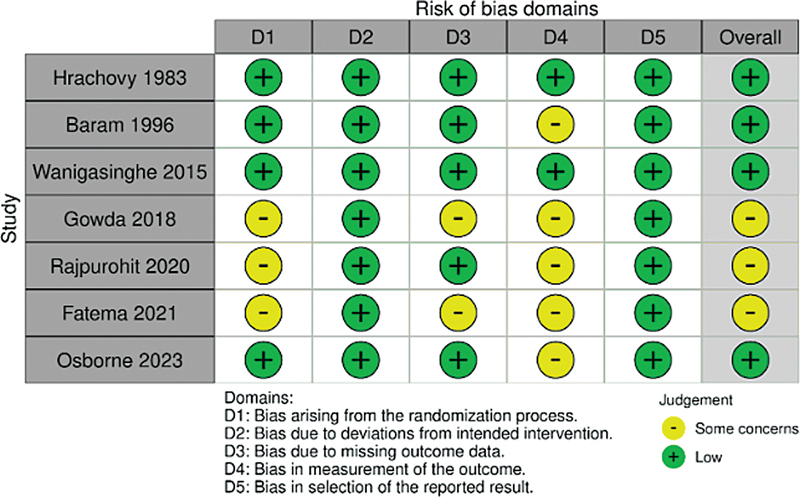
Risk of bias assessment of the included studies was conducted using version 2 of the Cochrane RoB2.

The assessment of the methodological quality of the included studies is summarized in Figure 4 and **Supplementary Material Figure S1** (online only). Most studies were classified as having an overall low risk of bias. However, three studies—Gowda et al., 2 Fatema et al., 12 and Rajpurohit et al. 4 —were identified as having some concerns in specific domains.

Gowda et al. 2 and Rajpurohit et al. 4 presented limitations in the randomization process (Domain 1), while Fatema et al. 12 and Rajpurohit et al. 4 showed concerns related to outcome measurement (Domain 4). Additionally, Rajpurohit et al. 4 demonstrated a combination of concerns across multiple domains, contributing to a higher overall risk of bias classification. Despite these issues, most studies exhibited adequate methodological designs, minimizing the potential impact of bias on the aggregated results of the present review.

## DISCUSSION

A meta-analysis conducted by Chang et al. 13 involved five RCTs, two of which are also included in the present analysis. They had a total of 239 participants, and the comparative efficacy of corticosteroids and ACTH in controlling infantile spasms associated with WS was investigated. The results did not reveal a statistically significant difference between the treatments in terms of clinical cessation of spasms (OR: 0.54; 95% CI: 0.16–1.81). Additionally, comparisons between high and low doses of prednisolone in ACTH also failed to demonstrate the superiority of either intervention.

Li et al. 14 in another meta-analysis that shares three studies with the present work, evaluated the cessation of spasms on day 14 and concluded that ACTH/tetracosactide is not superior to prednisolone/prednisone (RR: 1.19; 95% CI: 0.74–1.92). However, the analysis revealed substantial heterogeneity (I ^2^ = 67%). From an economic perspective, corticosteroids were associated with greater cost-effectiveness compared with ACTH, which, despite similar efficacy on day 14, incurs significantly higher costs. 15 These findings are reinforced by the results of the present meta-analysis, which incorporated two additional studies and demonstrated no superiority of ACTH or corticosteroids in controlling spasms during this period. 11

The robustness of the estimates was supported by sensitivity analyses, which confirmed consistency after sequential exclusions. The moderate heterogeneity identified did not compromise the overall interpretation of the data, and the influence analysis revealed minimal impact from most studies, with only one showing slight influence on the global results.

Regarding adverse effects, weight gain was highlighted as a common one, associated with both corticosteroids and ACTH. This gain is more pronounced in continuous therapies, while short-term treatments have a limited impact. 16 Another meta-analysis involving three RCTs did not demonstrate statistical significance regarding weight gain (RR: 0.86; 95% CI: 0.56–1.32; I ^2^ = 0%).

However, the present study, including three RCTs, found greater overall weight gain in the group treated with ACTH, with low heterogeneity (I ^2^ = 0%). This discrepancy may be attributed to the limited number of available studies, which restricts broader analyses and contributes to divergences regarding the actual impact of ACTH on this outcome. The presence of hypsarrhythmia was another variable investigated. In the literature, the absence of significant differences between ACTH and corticosteroids in this context is commonly reported. Li et al. 14 analyzed four RCTs and found no significant differences (RR: 1.14; 95% CI: 0.71–1.81; I ^2^ = 79%), although their study's high heterogeneity limits the generalization of the results. Our analysis included only one of the studies from their meta-analysis, and the findings reinforce the need for cautious interpretation due to heterogeneity (I ^2^ = 78%). However, a clinical trial evaluating hypsarrhythmia severity using a specific scale demonstrated significant improvement in the group treated with prednisolone compared with ACTH, suggesting that corticosteroids may offer additional benefits when assessed with detailed metrics.

The analysis of adverse effects also included hypertension, irritability, and infections. There were no significant differences between ACTH and corticosteroids regarding hypertension, with low heterogeneity, consistent with the findings of Chang et al. (OR: 1.19; 95% CI: 0.45–3.15; I ^2^ = 0%). 13 However, the present study identified a higher tendency toward hypertension in the ACTH (28 patients) compared with the corticosteroid group (12 patients), diverging from previous literature. Some studies included in the literature have also reported a higher frequency of adverse effects, such as arterial hypertension and cushingoid features, in patients treated with ACTH compared with those who received oral prednisolone.

The systematic review by Wanigasinghe et al. demonstrated that although both treatments show similar efficacy in the management of infantile spasms, ACTH was associated with a higher incidence of these adverse effects. 1 This difference in safety profiles should be considered when choosing a therapeutic strategy and further investigated in future randomized clinical trials. Irritability was more prevalent in the prednisolone group in Wanigasinghe et al., 1 Li et al., 14 and Chang et al. 13 did not find significant differences in this parameter. Regarding infections, while the ACTH group showed a tendency towards higher frequency, the difference was not significant, with moderate heterogeneity (I ^2^ = 56%). Paprocka et al. 16 identified infections as the main adverse effects of ACTH, including respiratory, gastrointestinal, and urinary.

Despite its contributions to the field, the present meta-analysis has limitations. The heterogeneity present in some of the analyses is an important limitation of this study. The included studies used different types and doses of corticosteroids, presented populations with slightly different clinical characteristics and adopted nonuniform methods for measuring the primary outcome. Additionally, two studies were conducted with an open design, which may have introduced detection and performance biases. These factors contribute to significant methodological heterogeneity, which should be considered when interpreting the results of the meta-analysis.

In summary, in a cohort of WS patients, both therapies showed similarity in terms of hypsarrhythmia and spasms control on day 14. There is a significant increase in weight gain with ACTH as compared with corticotherapy. Nevertheless, this hormone had a similar rate to all the other adverse events. These findings suggest that ACTH shouldn't be considered unanimously for the treatment of WS, since our study hasn't found significant benefits of adrenocorticotropic hormone compared with corticoids. Randomized controlled trials with a higher number of patients are warranted to elucidate if any approach should be the mainstay for WS, as well as to identify the nuances that determine whether ACTH or corticoids are more or less indicated to address infantile spasms syndrome, considering the individuality of each patient.
